# Downregulation of HMGB1 carried by macrophage-derived extracellular vesicles delays atherosclerotic plaque formation through Caspase-11-dependent macrophage pyroptosis

**DOI:** 10.1186/s10020-023-00753-z

**Published:** 2024-03-16

**Authors:** Weijie Liang, Ruibin Wei, Xingxing Zhu, Jinliang Li, Aiwen Lin, Jun Chen, Wen Wu, Qiang Jie

**Affiliations:** 1 Department of Cardiology, Panyu Central Hospital, Cardiovascular Institute of Panyu District, No. 8, Fuyu East Road, Qiaonan Street, Panyu District, Guangzhou, 511400 Guangdong Province People’s Republic of China; 2 Department of Endocrinology, Guangdong Geriatrics Institute, Guangdong Academy of Medical Sciences, Guangdong Provincial People’s Hospital, No. 106, Zhongshan Second Road, Yuexiu District, Guangzhou, 510080 Guangdong Province People’s Republic of China

**Keywords:** Atherosclerosis, Plaque formation, Extracellular vesicles, Macrophages, High-mobility group box 1, Foam cells, Pyroptosis, Inflammation

## Abstract

**Background:**

Macrophage-derived extracellular vesicle (macrophage-EV) is highly studied for its regulatory role in atherosclerosis (AS). Our current study tried to elucidate the possible role of macrophage-EV loaded with small interfering RNA against high-mobility group box 1 (siHMGB1) affecting atherosclerotic plaque formation.

**Methods:**

In silico analysis was performed to find critical factors in mouse atherosclerotic plaque formation. EVs secreted by RAW 264.7 cells were collected by ultracentrifugation and characterized, followed by the preparation of macrophage-EV-loaded siHMGB1 (macrophage-EV/siHMGB1). ApoE^−/−^ mice were used to construct an AS mouse model by a high-fat diet, followed by injection of macrophage-EV/siHMGB1 to assess the in vivo effect of macrophage-EV/siHMGB1 on AS mice. RAW264.7 cells were subjected to ox-LDL, LPS or macrophage-EV/siHMGB1 for analyzing the in vitro effect of macrophage-EV/siHMGB1 on macrophage pyrophosis and inflammation.

**Results:**

In silico analysis found that HMGB1 was closely related to the development of AS. Macrophage-EV/siHMGB could inhibit the release of HMGB1 from macrophages to outside cells, and the reduced HMGB1 release could inhibit foam cell formation. Besides, macrophage-EV/siHMGB also inhibited the LPS-induced Caspase-11 activation, thus inhibiting macrophage pyroptosis and preventing atherosclerotic plaque formation.

**Conclusion:**

Our results proved that macrophage-EV/siHMGB could inhibit foam cell formation and suppress macrophage pyroptosis, finally preventing atherosclerotic plaque formation in AS mice.

## Introduction

Atherosclerosis (AS) is a chronic vascular disease that causes cardiovascular disorders and is the leading cause of mortality and loss of productive life years globally (Herrington et al. 2016). Specifically, disease progression results in the formation of atherosclerotic plaques that lead to the narrowing of the arterial lumen and the formed atherosclerotic plaques often remain stable for years but can rapidly become unstable, rupture and induce thrombus formation (Emini Veseli et al. 2017; Lu et al. 2022). Accumulating evidence has documented the high plasma levels of low-density lipoprotein (LDL) as one of the most vital risk factors of AS, and LDL may accumulate in the sub-endothelial space of the arterial wall and subsequently forms oxidized LDL (oxLDL), which may trigger an inflammatory response (Tabas et al. 2015). Notably, atherosclerotic plaque formation is one of the typical pathological changes in AS and endothelial damage is confirmed as the critical pathophysiological mechanism (Gao et al. 2022). Vascular inflammation is partly caused by an influx of plasma LDL within the intima, where it is easy to be affected by oxidative modification, thus resulting in the recruitment of monocytes, which may differentiate into macrophages (Bouchareychas et al. 2020). The existing published literature has proposed that macrophages have an essential role in forming plaques, local inflammation, and thrombosis in AS (Back et al. 2019), so it's crucial to understand the molecular pathways in macrophages to prevent and treat AS.

Macrophages are the primary innate immune cells that have a wide range of activities in response to various infections or stimuli, and they play an essential role in the pathophysiology of many illnesses (Barrett 2020). Macrophage-derived small vesicles secreted by living cells are known as macrophage-derived extracellular vesicles (macrophage-EVs), which work as important messengers that can transfer different bioactive chemicals from macrophages to recipient cells. Thus macrophage-EVs are deemed mediators in the pathophysiology of various illnesses, including inflammatory disorders and fibrosis (Wang et al. 2020). Moreover, the critical role of macrophage-released exosomes in modulating AS has been characterized (Bouchareychas et al. 2020). Through bioinformatics analysis, we identified HMGB1 as the study subject.

Interestingly, HMGB1 has been reported to be a cargo of EVs (Willis et al. 2022), in line with our bioinformatics prediction. Further consultation of the literature displayed that HMGB1, a DNA-binding cytokine expressed mainly by macrophages, is a nuclear protein involved in the progression of AS and even some cardiovascular diseases (de Souza et al. 2012, Moreno et al. 2013, Zhang and Fernandez-Hernando 2021). Pyroptosis is a type of programmed cell death that occurs when the cysteinyl aspartate specific proteinase 1 (Caspase-1) and Caspase-4/5/11 enzymes are activated, and pyroptosis in macrophages is thought to be linked to atherosclerotic plaque formation (Yang et al. 2022). Additionally, the TNF-α/HMGB1 inflammation signaling pathway bears excellent responsibility in pyroptosis during acute kidney injury and acute liver failure (Wang et al. 2020). However, the detailed mechanism underlying HMGB1 in pyroptosis in AS remains to be further studied. Thus, we studied the effect of silenced HMGB1 loaded in macrophage-EVs on macrophage pyroptosis and atherosclerotic plaque formation.

## Materials and methods

### Bioinformatics analysis

The dataset used in this study was obtained from the GEO database (https://www.ncbi.nlm.nih.gov/geo/). The mRNA expression profiles related to atherosclerosis in mice (GSE28783) were selected. Four untreated AS mice (GSM712678, GSM712679, GSM712680, and GSM712681) were assigned to the treatment group (referred to as "treat"), while four AS mice that showed symptom relief after miR-33 treatment (GSM712670, GSM712671, GSM712672, and GSM712673) were assigned to the control group (referred to as "control"). Macrophages from the atherosclerotic plaques in the mice's aortas were used for gene expression profiling. Gene ID conversion was performed using the annotation file GPL1261 on the sequencing platform. The Limma package (version: 3.40.2, http://www.bioconductor.org/packages/release/bioc/html/limma.html) in R software was used to analyze the differential expression of mRNA. The threshold for distinguishing differentially express.

The interaction network of target genes was obtained from the STRING database (https://string-db.org) and subsequently imported into Cytoscape software (version: 3.8.2, https://cytoscape.org/). The regulatory relationship network was constructed, and Cytoscape was used to analyze the results and apply conditional filtering to the network diagram. The degree and combined score values were represented by colors and shapes in the network diagram.

### Isolation and characterization of macrophage-EVs

Macrophage-EVs were isolated from the supernatant of RAW264.7 cell medium by differential centrifugation. Briefly, after RAW264.7 cells were cultured in EV-free medium containing fetal bovine serum (FBS) for 48 h, the cell supernatant was collected and successively centrifuged at 800*g* for 5 min to remove dead cells, 1500*g* for 15 min to remove cell debris, and 15,000*g* for 30 min to remove large EVs. The obtained supernatant was then ultra-centrifuged at 150,000*g* for 2 h. The isolated macrophage-EVs were identified and used for the experiments.

Identification of macrophage-EVs was carried out by transmission electronic microscope (TEM, H-7650, HITACHI, Japan). Nanoparticle Tracking Analysis (NTA) was performed to determine macrophage-EVs' size. This analysis automatically tracks and determines particle size based on Brownian motion and diffusion coefficient. The macrophage-EV was resuspended in 1 mL PBS, and the filtered PBS served as a control. The diluted macrophage-EV was then injected into the NanoSight LM10 instrument to measure the particle size at 23.75 ± 0.5 ℃ with the measurement duration as 60 s.

Identification of surface protein markers of EVs was performed through Western blot. The EVs were extracted using Triton X-100 lysis buffer containing a proteinase inhibitor cocktail (Merck, 539134). The lysis buffer consisted of 20 mM Tris–HCl (pH 7.4), 137 mM NaCl, 1% Triton X-100, 2 mM EDTA, and 10% glycerol (Xu et al. 2017). Quantitative analysis was done using the BCA Protein Assay Kit (P0028, Biyuntian, Shanghai). The following antibodies were used for EV protein immunoblotting: CD9 (ab92726, 1:2000, Rabbit, Abcam, UK), CD63 (ab217345, 1:1000, Rabbit, Abcam, UK), and Calnexin (ab133615, 1:2000, Rabbit, Abcam, UK). The experiments were repeated three times.

### Preparation of macrophage-EV carrying small interfering RNA against HMGB1 (siHMGB1)

siHMGB1 was loaded in macrophage-EV using Gene Pulser X Cell Electroporation System by electroporation technique. The macrophage-EV with a total protein concentration of 20 μg was mixed with 20 μg of siHMGB1 in 400 μL PBS (pH = 7.3), which was immediately moved into the ice after 400 V electroporation. Unloaded siHMGB1 was removed by ultra-centrifugation at 100,000*g* for 1 h, followed by removing the supernatant. The precipitated macrophage-EV was resuspended, and the final prepared macrophage-EV loaded with siHMGB1 was called macrophage-EV/siHMGB1, with the sequence of the plasmid as siNC (5′-CAAAGACGACCAGGCGTATCGATCT-3′), siHMGB1 (5′-CAAGAAGAAGCACCCGGATGCTTCT-3′). The plasmids required for the experiment were purchased from GenePharma (Shanghai, China).

### Uptake of macrophage-EV/siHMGB1

PKH67 dye was added to macrophage-EV/siHMGB1 according to the kit instructions (PKH67GL, Sigma Aldrich, St. Louis, MO, USA), which was incubated at room temperature for 15 min, and then centrifuged at 1000*g* for 5 min, followed by removal of the supernatant. The mixture was suspended in a macrophage-EV medium and centrifuged at 1000 for 5 min. Precipitate was obtained after repeating twice, which was PKH67-labeled macrophage-EV. Then, RAW264.7 cells were incubated in the dish pre-coated with the specific cell slides. When cell confluence reached 50%, PKH67-labeled macrophage-EV/siHMGB1 was added for incubation at 37 ℃ for 24 h. The cell slides were removed, washed three times in PBS, soaked with 4% paraformaldehyde for 30 min at room temperature, permeabilized with 2% Triton X-100 for 15 min, and then stained by 4′,6-diamidino-2-phenylindole (DAPI; 2 μg/mL, C1005, Beyotime) for 10 min. Fluorescence expression was observed by confocal microscopy.

With the similar method mentioned above, Cy3-HMGB1 was loaded to the macrophage-EV. Macrophage-EV/Cy3-siHMGB1 was then co-cultured with RAW264.7 cells for 1 h and fixed in 4% paraformaldehyde. The nuclei were stained with DAPI. Internalization of macrophage-EV by RAW264.7 cells was observed under confocal microscopy.

### Uptake of Dli-ox-LDL by macrophages

Cell-specific slides were placed in cell culture dishes, in which RAW264.7 cells were seeded and incubated overnight with or without macrophage-EV/siHMGB1. Subsequently, 10 μg/mL of Dli-ox-LDL was incubated with cells at 37 ℃ for 24 h. After that, the cells were slowly washed 3 times, soaked in 4% paraformaldehyde for 30 min at room temperature, permeabilized using 2% Triton X-100 for 15 min, and stained with DAPI (2 μg/mL, C1005, Beyotime) for 10 min. The expression of the fluorescence was observed under confocal microscopy.

### Establishment of AS mouse models

Eighteen ApoE^−/−^ male mice (Laboratory Animal Resources, Chinese Academy of Sciences, Beijing, China) aged 8–10 weeks were maintained under specific pathogen-free conditions at 26–28 ℃ and 50–65% humidity. All animal experiments were approved by the Animal Ethics Committee of Panyu Central Hospital (GT-IACUC202208181), Cardiovascular Institute of Panyu District.

A total of 12 ApoE^−/−^ mice were fed a high-fat diet (protein: 20%; fat: 40%; carbohydrate: 40%; cholesterol: 1.25%) for 12 consecutive weeks for the establishment of AS model. The remaining 6 ApoE^−/−^ mice were fed regular food as a control. Six mice subjected to AS modeling were randomly selected and injected with macrophage-EV/siHMGB (100 μg/mouse, once every two days) for 12 weeks, and the remaining mice were injected with PBS as a control.

### Cell culture and grouping

Mouse mononuclear macrophage RAW264.7 (CL-0190, Procell, Wuhan, China) were cultured in Dulbecco’s modified eagle medium (PM150210, Procell) with 100 U/mL penicillin and 100 U/mL streptomycin (PB180120, Procell) at 37 ℃, with 5% CO_2_. The culture medium was renewed every 3 days. When cell confluence reached 80%, cell passage was performed with 0.25% trypsin/ethylenediamine tetraacetic acid.

At 24 h post cell seeding in 6-well plates, RAW264.7 cells were treated with 20 μg/mL macrophage-EV, 20 μg/mL macrophage-EV/siHMGB, or 50 μg/mL ox-LDL for 48 h, or with 1 μg/mL LPS for 24 h. Cells treated with an equal volume of PBS were taken as the control.

### Enzyme-linked immunoassay (ELISA)

Serum and cell culture supernatants were collected for determination of the contents of HMGB1, TNF-α, IL-6, IL-1β, and IL-18 using the hum-HMGB1 (SP11733, Procell), mmu-HMGB1 (SP14752, Procell), TNF-α (SP13726, Procell), IL-6 (SP13755, Procell), IL-1β (SP13701, Procell), and IL-18 (SP13710, Procell) kits. The absorbance of each well was measured within 30 min using a microplate reader at 450 nm.

### Oil red o staining

Mouse aorta was taken from differently-treated mice, washed with precooled PBS, put into a small box with liquid-embedded gel, and quickly frozen in liquid nitrogen. The frozen tissue blocks were sliced to 5 μm thickness, and sections were stained by Oil red O staining. For the macrophages, cell slides were washed with precooled PBS, fixed with 4% paraformaldehyde for 30 min at room temperature, washed in 50% ethanol after drying, and treated with the Oil Red O Ethanol Dye Solution for 8 min. Then, the cell slides were differentiated by 50% ethanol, counterstained by hematoxylin, and washed with running water to return to blue color. After air-drying, the cell slides were mounted and observed under a light microscope to analyze the vascular atherosclerotic plaque. Plaque area was quantified using Image Pro Plus (IPP) software.

### RNA isolation and quantification

Total RNA was extracted using Trizol (16096020, Thermo Fisher Technology, New York, NY, USA). The purity and concentration of the obtained RNA were assessed by measuring the absorbance at 260 and 280 nm. Reverse transcription was performed according to the instructions of the complementary DNA reverse transcription kit (RR047A, Takara, Japan). Polymerase chain reaction (PCR) was performed according to the instructions of LightCycler 480 SYBR Green I Master (04707516001, Roche, Germany) with glyceraldehyde-3-phosphate dehydrogenase (GAPDH) used as the internal reference. The relative mRNA expression was detected with the 2^−ΔΔCt^ method, and the primer sequences are shown in Additional file 1: Table S1.

### Western blot analysis

Total protein was extracted from tissues, cells, and EVs using RIPA lysis buffer (P0013B, Beyotime) containing PMSF, and the protein concentration was determined using a BCA kit (P0028, Beyotime). Proteins were denatured by boiling at 100 ℃ for 10 min and then saved at – 80 ℃. According to the size of the target protein band, 8–12% sodium dodecyl-sulfate electrophoresis gels were prepared, and equal volumes of protein samples were added to each lane for electrophoresis separation. The protein from the gel was transferred to a polyvinylidene fluoride membrane (1620177, BIO-RAD, Hercules, CA, USA), which was blocked by 5% bovine serum albumin for 1 h at room temperature. The membrane was incubated with primary antibodies against HMGB1 (ab79823, 1:10,000, Rabbit, Abcam), Caspase-11 (ab180673, 1:1000, Rabbit, Abcam), LOX-1 (11837-1-AP, 1:1000, Rabbit, Proteintech), CD36 (18836-1-AP; 1:1000, Rabbit, Proteintech), SRA (ab151707, 1:1000, Rabbit, Abcam), FL-GSDMD (# 39754, 1:1000, Rabbit, CST, Danvers, MA, USA), and N-GSDMD (#39754, 1:1000, Rabbit, CST) overnight at 4 ℃. The next day, the membranes were incubated with HRP-labeled goat anti-rabbit IgG (ab6721, 1:5000, Abcam) for l h at room temperature. The membrane was immersed in an enhanced chemiluminescence reaction solution (1705062, Bio-Rad) at room temperature for 1 min and then developed on an Image Quant LAS 4000C gel imager. GAPDH (A01021, 1:5000, Rabbit, Abbkine, Wuhan, China) was used as an internal reference to detect the expression level of each protein.

### Immunohistochemistry (IHC)

Paraffin-embedded sections of the mouse aorta were dewaxed to water, dehydrated by gradient alcohol, and subjected to antigen retrieval. Sections were blocked with normal goat serum blocking solution (C0265, Beyotime) at room temperature for 20 min. Then, the sections were incubated with primary antibody HMGB1 (ab79823, 1:400, Rabbit, Abcam) at 4 ℃ overnight, followed by another incubation with the secondary antibody goat anti-rabbit IgG (ab6721, 1:1000, Abcam) at 37 ℃ for 20 min. Next, the sections were incubated with HRP-labeled Streptavidin (A0303, Beyotime) at 37 ℃ for 20 min and developed by DAB (P0203, Beyotime). Sections were counterstained by hematoxylin (C0107, Beyotime) for 1 min, treated with 1% ammonia to return to blue color, dehydrated, cleared by xylene and mounted. Subsequently, the sections were observed and photographed by a microscope. An experienced pathologist read the sections, and 5 high-power fields were randomly selected for analysis in each section. Each field counted 100 cells, and the percentage of positively stained cells was calculated.

IHC score = A × B (A: 0 indicates cells without positive staining, 1 indicates 10% positive cells, 2 indicates 11–50% positive cells, 3 indicates 51–80% positive cells and 4 indicates more than 80% positive cells. B: 0 indicates no staining, 1 indicates shallow staining intensity, 2 indicates medium staining intensity, and 3 indicates deep staining intensity).

### Immunofluorescence (IF)

Paraffin-embedded sections of the mouse aorta were taken, dewaxed, dehydrated, and subjected to antigen retrieval. Sections were added with 0.03% Triton for 10 min and sealed with normal goat serum blocking solution (C-0005, HaoranBio, Shanghai, China) for 60 min at room temperature. For macrophages, macrophages were fixed with 4% paraformaldehyde for 30 min, washed with precooled PBS And sealed as the above. Next, primary antibodies of HMGB1 (66525-1-Ig, 1:250, Mouse, Proteintech, VA, USA), F4/80 (ab6640, 1:50, Rabbit, Abcam), GSDMD (sc-393581, 1:200, Mouse, SANTA, UC, USA), iNOS (ab210823, 1: 100, Mouse, Abcam), and CD206 (60143-1-Ig, 1:200, Mouse, Proteintech) were selected for sample incubation at 4 ℃ overnight. Then, the samples were incubated with fluorescent secondary antibody, Alexa Fluor^®^ 647-Anti-Rabbit IgG (ab150075, 1:500, Donkey, Abcam) or Alexa Fluor^®^ 488-Anti-Mouse IgG (ab150113, 1:500, Goat, Abcam) at room temperature for 60 min in the dark. A fluorescence microscope was utilized for observation, with fluorescence intensity recorded.

### Flow cytometry

Cells were collected after different treatments and then made into single-cell suspensions. Next, cell suspension was incubated with F4/80-FITC (11-4801-82, eBioscience, San Diego, CA, USA), iNOS-PE (61-5920-82, eBioscience), and CD206-APC (17-2061-82, eBioscience). After incubation, cells were collected and analyzed using a CytoFlex flow cytometer (Beckman Coulter, Brea, CA, USA). Data were analyzed using the FlowJo software (version 7.0; FlowJo LLC, Ashland, OR, USA).

### Statistical analysis

Statistical analysis of the study data was performed using SPSS 21.0 (IBM, Armonk, NY, USA). Measurement data are expressed as mean ± standard deviation. The data conformed to normal distribution and homogeneity of variance. The *t*-test was applied for comparing data between groups, while one-way analysis of variance was for comparing data among multiple groups, followed by Tukey's post-hoc test. A *p* < 0.05 indicates a statistically significant difference.

## Results

### HMGB1 may influence the occurrence and development of AS

**Fig. 1:**
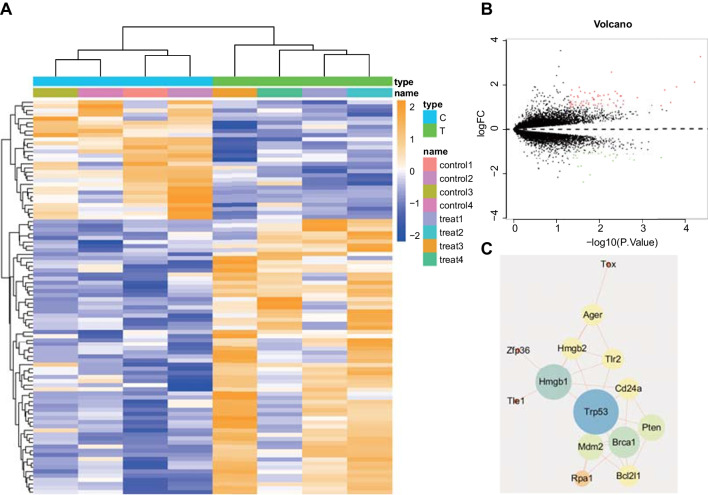
Screening of differentially expressed genes in AS through bioinformatics analysis. **A** Heatmap of differential gene expression in microarray dataset GSE28783 (control = 4; Treat = 4). **B** Volcano map of differential gene expression in microarray dataset GSE28783 (red represents upregulated genes and green represents downregulated genes). **C** PPI network map of candidate target genes (the more significant the shape is, the bigger the degree value is)

Using differential analysis of gene expression data from the GEO database on AS disease in mice, we analyzed the GSE28783 dataset. The treated group consisted of AS mice (GSM712678, GSM712679, GSM712680, and GSM712681), while the control group comprised AS mice with alleviated symptoms after treatment (GSM712670, GSM712671, GSM712672, and GSM712673). We obtained macrophages from atherosclerotic plaques in mouse aortas for differential analysis, resulting in 96 differentially expressed genes. The heatmap of gene expression is displayed in Fig. 1A, and the volcano map showing differential gene expression is shown in Fig. 1B. The target genes of differentially expressed candidates were imported into the String database to obtain the protein interaction relationship, which was further imported into Cytoscape software to construct a PPI network. We predicted the top 10 hub genes as TRP53, HMGB1, BRCA1, PTEN, MDM2, AGER, TLR2, HMGB2, BCL2L1, and CD24A through the MCC network of cytoHubba (Fig. 1C). Previous literature has reported that HMGB1 may promote the occurrence and progression of various AS-related diseases such as pulmonary hypertension, coronary heart disease and ischemic stroke by inducing the infiltration of leukocytes, adhesion aggregation of platelets and the migration of vascular smooth muscle cells (Ding et al. 2016). Therefore, HMGB1 was selected as the target gene in this study.

### Increased HMGB1 expression and accumulated macrophage infiltration were found in the plaque tissue of AS mice

HMGB1 is the most abundant DNA binding protein in the nucleus, which is mainly expressed in macrophages and can be released outside of cells under pressure, thus promoting the development of AS (Ghaffari et al. 2021). Therefore, we next investigated the molecular mechanisms underlying the effect of HMGB1 expression in macrophages on AS progression.

**Fig. 2:**
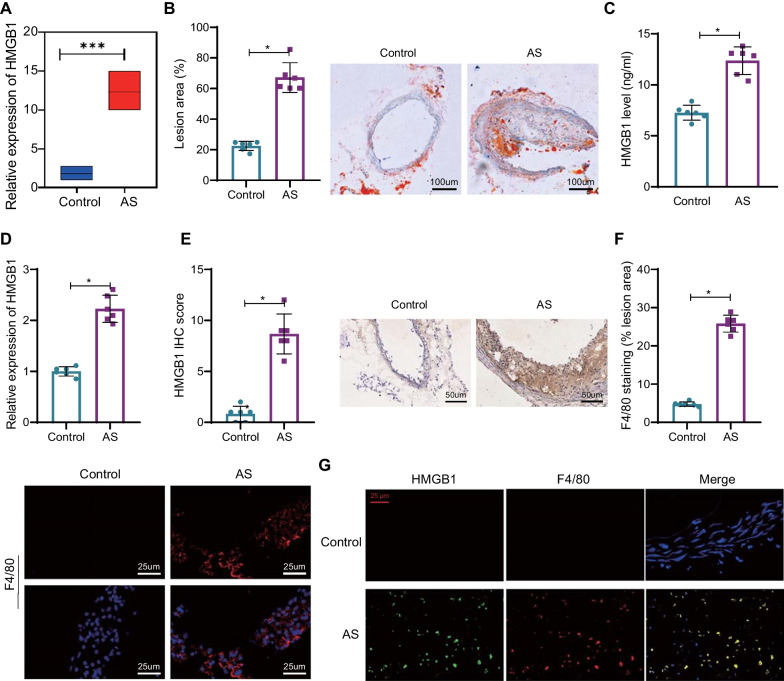
Upregulated HMGB1 expression and increased macrophage infiltration in AS. **A** Box-plot of HMGB1 expression in the GSE28783 dataset (control = 4; AS = 4). **B** The size of atherosclerotic plaques at the aortic root determined by oil red O staining. **C** Protein levels of HMGB1 in mouse serum measured by ELISA. **D** HMGB1 expression in the mouse aortic root detected by RT-qPCR. **E** Protein expression of HMGB1 in atherosclerotic plaque tissue determined by IHC. **F** Expression of F4/80 in atherosclerotic plaque tissues studied by IF. **G** IF detection of co-localization of HMGB1 and macrophages in atherosclerotic plaque tissue (400 × , 25 μm). Red fluorescence represents the F4/80 positive Expression, while green fluorescence represents the positive Expression of HMGB1. ***Indicates *p* < 0.001; *indicates *p* < 0.05. n = 6. Data between the two groups were compared using an independent sample *t*-test

Further analysis of HMGB1 expression in the microarray dataset GSE28783 showed that HMGB1 expression was significantly higher in AS samples (Fig. 2A). The ApoE^−/−^ mice were fed a high-fat diet to induce AS. As displayed by Oil red O staining, atherosclerotic plaque in AS mice was significantly more prominent than in control mice (Fig. 2B), highlighting the successful establishment of AS models. Besides, the protein level of HMGB1 in the serum of AS mice was higher than that in control mice (Fig. 2C). HMGB1 expression in the aortic root was validated to be higher in AS mice, as measured by RT-qPCR and IHC (Fig. 2D, E). Suppose the expression of F4/80 in atherosclerotic plaques was detected to determine the macrophage infiltration in AS. The results showed that more macrophage infiltration was found in AS mice (Fig. 2F). Also, IF results revealed that in AS plaque tissues, the green fluorescent-labeled HMGB1 overlapped with red fluorescent-labeled F4/80, which showed that HMGB1 was expressed in macrophages (Fig. 2G).

The above results showed that the macrophage infiltration increased, and the expression of HMGB1 was upregulated in the plaque tissues of AS mice. Therefore, we speculated that altered expression of HMGB1 in macrophages could prevent atherosclerotic plaque formation.

### Macrophage-EV/siHMGB1 inhibits HMGB1 expression in macrophages

Macrophage-EV is a lipid bilayer heterogeneous particle of 20 nm to 2 µm size involved in AS-related processes such as endothelial dysfunction, vascular wall inflammation, and remodeling (Wu et al. 2020). Therefore, macrophage-EV/siHMGB1 was applied for the treatment of AS.

**Fig. 3:**
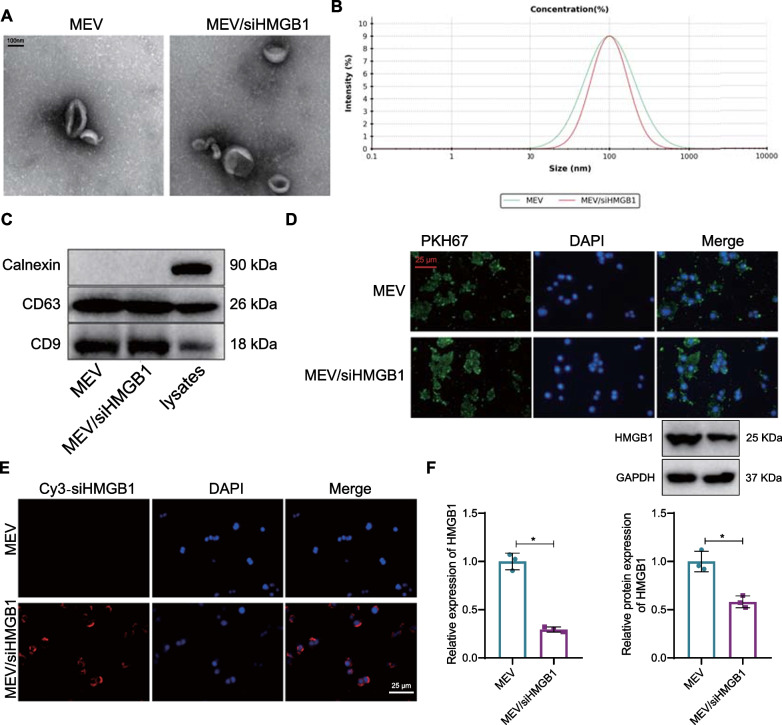
Preparation and identification of macrophage-EV/siHMGB1. **A** The morphology of macrophage-EV and macrophage-EV/siHMGB1 observed by TEM (100 nm). **B** Diameter size of macrophage-EV and macrophage-EV/siHMGB1 measured by NTA. **C** CD63, CD9, and Calnexin content on EV surface detected by Western blot analysis. **D** Uptake of macrophage-EV and macrophage-EV/siHMGB1 in RAW264.7 cells observed by laser confocal microscope (400 × , 25 μm). The PKH67-labeled macrophage-EV and macrophage-EV/siHMGB1 showed green fluorescence. **E** Uptake of siHMGB1 by RAW264.7 cells analyzed by laser confocal microscope (400 × , 25 μm). The cy3-labeled siHMGB1 shows red fluorescence. **F** HMGB1 expression detected by RT-qPCR and Western blot after RAW264.7 cells were treated with macrophage-EV and macrophage-EV/siHMGB1. *Iindicates *p* < 0.05. Data between the two groups are compared using an independent sample t-test. Data among multiple groups are compared using one-way ANOVA, followed by Tuckey's post hoc test

RAW264.7 cell-derived EVs were isolated, and siHMGB1 was loaded into the macrophage-EV by electroporation. TEM results showed that the morphology of macrophage-EV and macrophage-EV/siHMGB1 was uniform round or oval membranous vesicle-like (Fig. 3A). NTA results displayed that the average particle diameter of macrophage-EV and macrophage-EV/siHMGB1 was 100 nm (Fig. 3B). The EV marker proteins CD63 and CD9 were significantly highly expressed in the macrophage-EV and macrophage-EV/siHMGB1 surface. In contrast, no endoplasmic reticulum protein Calnexin was detected (Fig. 3C). The above results confirmed that the isolation of macrophage-EV was successful. Macrophage-EV/siHMGB1 had the same characteristics as macrophage-EV.

PKH67-labeled macrophage-EV and macrophage-EV/siHMGB1 were co-cultured with RAW264.7 cells, respectively. After 24 h, the Uptake of macrophage-EV and macrophage-EV/siHMGB1 by RAW264.7 cells was observed. Laser confocal microscope results showed that macrophage-EV and macrophage-EV/siHMGB1 could be uptaken by RAW264.7 cells (Fig. 3D). Moreover, macrophage-EV/siHMGB1 could deliver siHMGB1 to RAW264.7 cells (Fig. 3E). The expression of HMGB1 in RAW264.7 cells co-cultured with macrophage-EV/siHMGB1 was reduced (Fig. 3F). The results indicated that macrophage-EV/siHMGB1 could be effectively taken up by macrophages and inhibit the expression of HMGB1 in macrophages.

### Macrophage-EV/siHMGB1 prevents plaque formation in AS mice

**Fig. 4:**
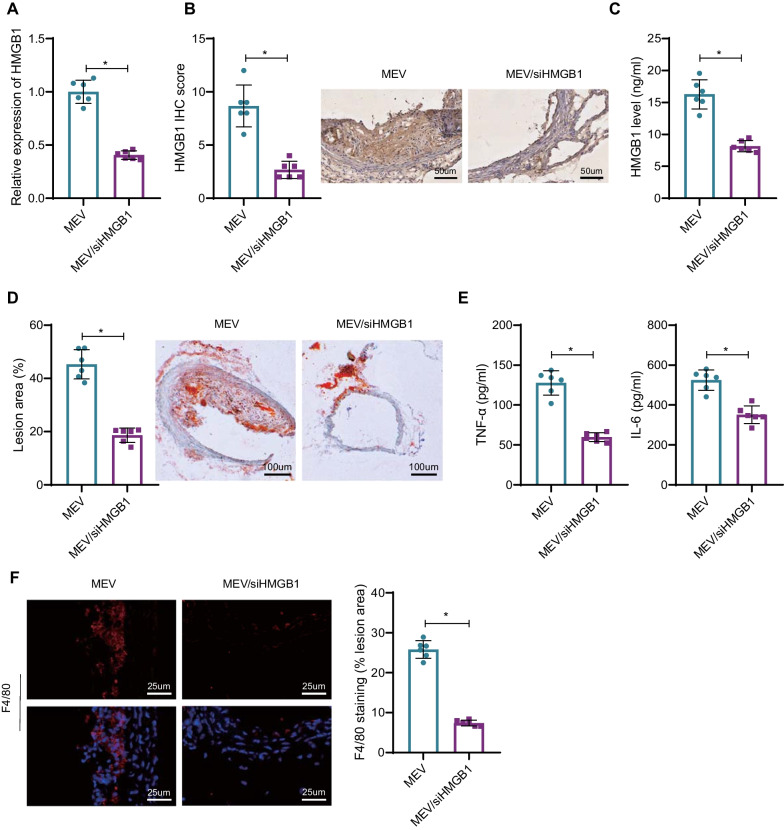
Macrophage-EV/siHMGB1 inhibits atherosclerotic plaque formation in mice. **A** HMGB1 expression in atherosclerotic plaque tissue of AS mice following macrophage-EV/siHMGB1 detected by RT-qPCR. **B** Expression of HMGB1 in the atherosclerotic plaque tissues of the mouse thoracic aorta assessed by IHC. **C** Protein content of HMGB1 in mouse serum measured by ELISA. **D** Atherosclerotic plaque size in the thoracic aorta determined by Oil red O staining. **E** Levels of inflammatory cytokines TNF-α and IL-6 in mice serum measured by ELISA. **F** Expression of F4/80 in atherosclerotic plaque tissue detected by IF. *Indicates *p* < 0.05. n = 6. Data between the two groups were compared using an independent sample *t*-test

AS mice were treated with or without macrophage-EV/siHMGB1 to observe the effect of macrophage-EV/siHMGB1 on atherosclerotic plaque formation in mice. First, both HMGB1 mRNA and protein levels were decreased after injection of macrophage-EV/siHMGB1 relative to macrophage-EV (Fig. 4A). IHC staining was performed to detect the expression of HMGB1 in atherosclerotic plaque tissue of mouse thoracic aorta. The results revealed decreased expression of HMGB1 in the MEV/siHMGB1 group (Fig. 4B). Moreover, ELISA results showed that relative to macrophage-EV, the protein content of HMGB1 in serum of AS mice after macrophage-EV/siHMGB1 injection was also reduced (Fig. 4C).

In addition, relative to macrophage-EV, after injection of macrophage-EV/siHMGB1 in AS mice, atherosclerotic plaques were distinctly reduced (Fig. 4D), while TNF-α and IL-6 levels in mouse serum were lower (Fig. 4E). Also, as IF detected, macrophage-EV/siHMGB1 could curtail macrophage infiltration in atherosclerotic plaque tissues (Fig. 4F).

The above results demonstrated that macrophage-EV/siHMGB1 effectively prevented plaque formation in AS mice.

### Macrophage-EV/siHMGB1 suppresses the release of HMGB1 to prevent the formation of foam cells and inflammatory response in macrophages

**Fig. 5:**
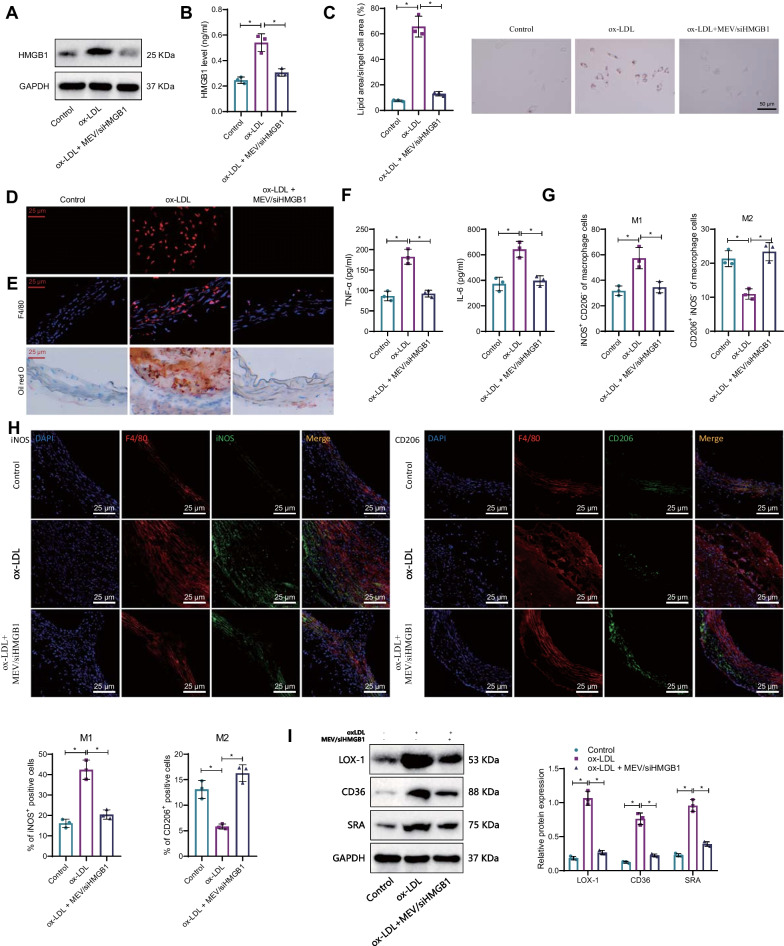
Macrophage-EV/siHMGB1 suppresses the release of HMGB1 expression to inhibit foam cell formation and inflammatory responses in macrophages. **A** Expression of HMGB1 in macrophages treated with ox-LDL and intervened with MEV/siHMGB1 was detected by Western blotting. **B** HMGB1 content in the macrophage culture supernatants after macrophage-EV/siHMGB1 or ox-LDL treatment measured by ELISA. **C** Lipid droplet in macrophages to reflect foam cell formation analyzed by Oil red O staining. **D** Uptake of Dli-labeled ox-LDL by macrophages determined by IF (400 × , 25 μm). **E** Foam cell formation in plaque tissues of AS mice after macrophage-EV/siHMGB1 treatment assessed by Oil red O and F4/80 staining (400 × , 25 μm). **F** Levels of TNF-α and IL-6 in the macrophage culture supernatants studied by ELISA. **G** Macrophage polarization measured by flow cytometry. **H** Macrophage polarization in the plaque tissues of AS mice tracked by IF. **I** Expression of LOX-1, SR-A, and CD36 in macrophages treated with ox-LDL and intervened with MEV/siHMGB1 by Western blot analysis. iNOS is the M1-type macrophage marker, while CD206 is an M2-type macrophage marker. *Indicates *p* < 0.05. Data among multiple groups are compared using one-way ANOVA, followed by Tuckey’s post hoc test

We further investigated the molecular mechanism of macrophage-EV/siHMGB1 in preventing atherosclerotic plaque formation in vitro. As documented, plaque accumulation in the AS vessel wall is associated with macrophage uptake of LDL and the conversion of macrophages into foam cells (Tao et al. 2021). Therefore, we used oxidized LDL to stimulate macrophages and observed the effect of macrophage-EV/siHMGB1 on the conversion of macrophages into foam cells. We found that the protein content of HMGB1 in macrophages increased after ox-LDL stimulation but increased in the supernatants of cell culture medium, and the protein content of HMGB1 in both macrophages and cell culture medium supernatants was decreased after the addition of macrophage-EV/siHMGB1 (Fig. 5A, B). These results elaborated that macrophage-EV/siHMGB1 could inhibit HMGB1 protein expression in macrophages and reduce its extracellular release.

Oil-red O staining was applied to measure macrophage lipid droplet content to reflect foam cell formation. The results showed that ox-LDL stimulation promoted foam cell formation, but it was suppressed by macrophage-EV/siHMGB1 (Fig. 5C). Uptake of Dli-labeled ox-LDL by macrophages was analyzed by IF, which showed that macrophage-EV/siHMGB1 inhibited ox-LDL uptake by macrophages (Fig. 5D). Furthermore, foam cells increased in plaques of AS mice, but then decreased after addition of macrophage-EV/siHMGB1 (Fig. 5E). The above results concluded that macrophage-EV/siHMGB1 could inhibit the Uptake of ox-LDL by macrophages and then inhibit foam cell formation.

Macrophage inflammation is involved in AS's development (Tao et al. 2021). Besides, HMGB1 can promote the inflammatory response in macrophages (Yan et al. 2021). Therefore, we further investigated the effect of macrophage-EV/siHMGB1 on macrophage inflammation. ELISA results showed that ox-LDL stimulation promoted the secretion of TNF-α and IL-6 by macrophages, while those were reduced by macrophage-EV/siHMGB1 (Fig. 5F).

Flow cytometry results showed that the proportion of M1-polarized macrophages was significantly higher than that of M2-polarized macrophages after ox-LDL stimulation, while macrophage-EV/siHMGB1 led to opposite trends (Fig. 5G).

IF detection showed that the proportion of iNOS^+^ cells in plaque tissues of AS mice was significantly higher than that of CD206^+^ cells, while macrophage-EV/siHMGB1 caused opposite trends (Fig. 5H).

To investigate the changes in scavenger receptors during foam cell formation, we employed western blot analysis to examine the protein expression of LOX-1, CD36, and SRA in the plaque tissue of mice. The results indicated that macrophages exhibited upregulation of scavenger receptors LOX-1, CD36, and SRA following ox-LDL treatment compared to the control group. However, treatment with MEV/siHMGB1 attenuated the upregulation of these receptors (Fig. 5I).

Collectively, MEV/siHMGB1 inhibits HMGB1 protein expression and extracellular release in macrophages, promotes M2 polarization of macrophages, and downregulates the expression of scavenger receptors LOX-1, CD36, and SRA, thereby inhibiting foam cell formation and macrophage inflammatory response, ultimately preventing the formation of AS plaques.

### Macrophage-EV/siHMGB1 inhibits caspase-11-dependent macrophage pyroptosis to prevent atherosclerotic plaque formation

Cell death and inflammation are two vital pathological mechanisms of AS, and pyroptosis of macrophages can promote the development of AS (Wu et al. 2018a, b; Jiang et al. 2021). There are two main ways of pyroptosis, classical and nonclassical pyroptosis, and activated Caspase-11 in the noncanonical pathway cleaves the linker region of full-length GSDMD (FL-GSDMD) to separate N-GSDMD, which selectively interacts with membrane lipids to form transmembrane pores through which cellular contents are released substances such as IL-1β and IL-18, which initiate pyroptosis (Burdette et al. 2021). Therefore, we further investigated the effect of macrophage-EV/siHMGB1 on macrophage pyroptosis.

**Fig. 6:**
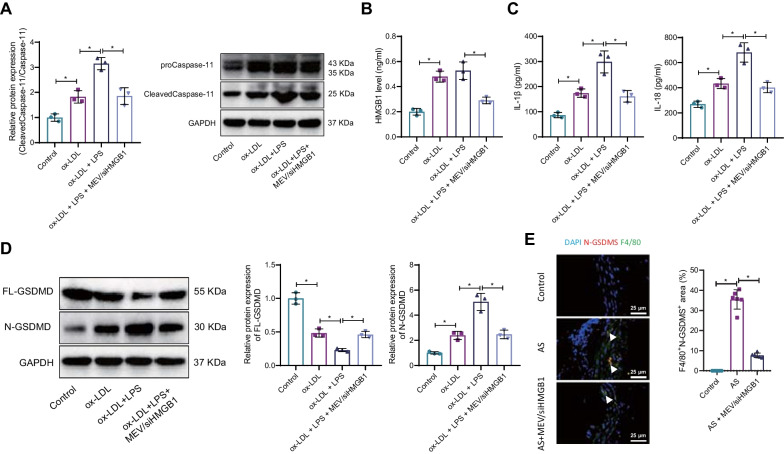
Macrophage-EV/siHMGB1 inhibits Caspase-11-dependent macrophage pyroptosis to prevent atherosclerotic plaque formation. **A** Western blot analysis detected the Activation of Caspase-11 in macrophages following ox-LDL, LPS, or macrophage-EV/siHMGB1 treatment. **B** HMGB1 Expression in the cell culture supernatants following ox-LDL, LPS, or macrophage-EV/siHMGB1 treatment measured by ELISA. **C** Expression of pyroptosis-related inflammatory factors IL-1β and IL-18 in cell culture medium supernatants following ox-LDL, LPS, or macrophage-EV/siHMGB1 treatment determined by ELISA. **D** Expression of the pyroptosis-related proteins FL-GSDMD and N-GSDMD in cells following ox-LDL, LPS, or macrophage-EV/siHMGB1 treatment measured by Western blot analysis. **E** Pyroptosis in the aortic root of AS mice following macrophage-EV/siHMGB1 treatment studied by IF. *Indicates *p* < 0.05. n = 6. Data among multiple groups are compared using one-way ANOVA, followed by Tuckey’s post hoc test

It is reported that HMGB1 can activate Caspase-11 by extracellular LPS and then induce macrophage pyroptosis (Deng et al. 2018). Thus, we used LPS stimulation of macrophages to mimic the inflammatory environment in AS to observe the activation of Caspase-11/Caspase-11 by macrophage-EV/siHMGB1.Western blot results showed that ox-LDL could promote the expression of Cleaved-Caspase-11/Caspase-11 in macrophages, and LPS stimulation led to further increase in the expression of Cleaved-Caspase-11/Caspase-11, but macrophage-EV/siHMGB1 intervention inhibited Cleaved-Caspase-11/Caspase-11 expression (Fig. 6A). As measured by ELISA, the ox-LDL stimulation increased content of HMGB1, while there was no significant difference in HMGB1 content after LPS stimulation. The increase of HMGB1 was inhibited after adding macrophage-EV/siHMGB1 (Fig. 6B).

Cell pyroptosis-related inflammatory cytokines IL-1β and IL-18 were upregulated by ox-LDL, which were further increased after LPS stimulation. But the elevation was suppressed after macrophage-EV/siHMGB1 intervention (Fig. 6C). The expression of pyroptosis-related proteins FL-GSDMD and N-GSDMD was analyzed by Western blot analysis. After ox-LDL stimulation, N-GSDMD expression increased, while FL-GSDMD expression decreased, and N-GSDMD expression further increased after LPS stimulation, along with reduced FL-GSDMD expression. The results of Western blot analysis revealed that compared to the control group, the expression of N-GSDMD was increased in the ox-LDL group, while the expression of FL-GSDMD was decreased. Additionally, upon stimulation with LPS, the expression of N-GSDMD further increased, while the expression of FL-GSDMD decreased in macrophages. However, the intervention with MEV/siHMGB1 attenuated the effects of both ox-LDL and LPS interventions (Fig. 6D). Moreover, the number of F4/80^+^N-GSDMS^+^ cells was significantly higher in AS mice but then reduced by macrophage-EV/siHMGB1 (Fig. 6E).

The above results confirmed that macrophage-EV/siHMGB1 could inhibit the release of HMGB1 from macrophages and then inhibit LPS-activated Caspase-11-dependent pyroptosis, subsequently preventing atherosclerotic plaque formation.

## Discussion

Macrophage-EVs have emerged as vital modulators in diverse diseases and cancers (Wang et al. 2020). Moreover, macrophage-EVs can load siRNA targeting genes, such as chemokine receptors, to exert suppressive function on inflammation and thus relieve septic symptoms (Ding et al. 2022), which offers functional evidence for the successful therapeutic targeting of macrophage-EVs, ratifying the clinical development of gene inhibition. Our current study showed macrophage-EVs could load siHMGB1 to reduce HMGB1 expression, prevent plaque formation, and relieve inflammatory response in macrophages, thus alleviating AS.

Through our bioinformatics analysis, we found that HMGB1 might influence the progression of AS, and our further experimental results unfolded that HMGB1 was highly expressed in AS samples, which was expressed in macrophages. It has been reported that damage to endothelial cells leads to the attraction of macrophages, which is critical for the development of AS, and the development of AS is accompanied by a prolonged pro-inflammatory response (Kake et al. 2020). Accumulating evidence has found the expression of HMGB1 and RAGE in endothelial cells, smooth muscle cells, and macrophages of atherosclerotic lesions (Kalinina et al. 2004, Naglova and Bucova 2012). Specifically, up-regulation of HMGB1 is capable of causing the intensification of inflammatory response in endothelium lesions and thus accelerating further atherosclerotic changes (Zhang and Fernandez-Hernando 2021). Consistent with our results, HMGB1 was upregulated in the aorta tissues of atherosclerotic mouse models (Wang et al. 2020). Moreover, in ApoE^−/−^ mice, HMGB1 knockdown has decreased vascular inflammation and ameliorated AS (Liu et al. 2013). Interestingly, the regulatory function of HMGB1 in macrophages by metformin in AS has already been elucidated (Feng et al. 2021).

HMGB1 is a chromatin-binding protein that is universally present in the cell nucleus. It maintains chromosomal stability and regulates physiological processes such as DNA replication, transcription, and repair. It is also a member of the damage-associated molecular pattern (DAMP) family and can trigger the inflammatory response of immune cells (Keller et al. 2008; de Torre-Minguela et al. 2016; Tang et al. 2017; Khambu et al. 2019). During atherosclerosis, HMGB1 is released by activated macrophages, leading to their apoptosis and promoting the expression of other inflammatory factors. These inflammatory factors can recruit macrophages, forming a vicious cycle. This is believed to be one of the reasons for the formation of macrophage-derived foam cells induced by HMGB1 (Wu et al. 2018a; b). Studies have shown that HMGB1 promotes atherosclerosis by interacting with the TLR4 receptor, leading to the activation of NF-κB and the induction of inflammation (Wu et al. 2018a; b). Additionally, HMGB1 has been found to play a role in the development of atherosclerosis by promoting cell necrosis, but the specific mechanisms need further investigation. Overall, HMGB1 plays a significant role in the formation of atherosclerosis, potentially mediating inflammation and cell necrosis through the TLR4 signaling pathway.

Due to their inherent characteristics, EVs, nature's RNA carriers, are increasingly being investigated as alternative siRNA delivery vehicles (Evers et al. 2022). Therefore, we loaded siHMGB1 onto macrophage-EVs by electroporation. Then, the AS mouse model induced by a high-fat diet was injected with or without the successfully developed macrophage-EV/siHMGB1. We found that injection of macrophage-EV/siHMGB1 could relieve the inflammatory response in macrophages (as evidenced by reduced levels of TNF-α and IL-6) and dampen atherosclerotic plaque formation. TNF-α and IL-6 are the markers for the inflammatory effects of activated macrophages in AS (Liu et al. 2022). Macrophages in the artery wall can lead to an imbalance of macrophage recruitment to plaque, contributing to the inflammation in AS and plaque instability (Moore et al. 2013). In terms of transcription regulation, the transcription levels of IL-1β and IL-18 are controlled by multiple transcription factors, including NF-kappaB, AP-1, and C/EBP (Venkatesan et al. 2010; Maranto et al. 2011; Abbate et al. 2020; Mantsounga et al. 2022; Wan et al. 2022). These transcription factors are activated under specific conditions and bind to the IL-1β gene promoter region to facilitate IL-1β transcription. Additionally, inflammatory factors such as TNF-α and IL-6 can regulate the expression and release of IL-1β (Hirano et al. 2017; Batra et al. 2018). Overall, IL-1β and IL-18 are subject to regulation by various pathways. One possible explanation is that multiple regulatory mechanisms influence the production of IL-1β and IL-18 in a complex inflammatory environment. Therefore, a single siHMGB1 treatment alone is insufficient to completely reverse these effects, and thus, the treatment of ox-LDL-LPS + siHMGB1 is also inadequate to reduce these cytokines to the level of the Control group.

Furthermore, the key pathological features of AS contain macrophage infiltration and foam cell formation (Zong et al. 2022). As previously documented, HMGB1 depicts increased expression in nuclei and cytoplasm of macrophages and smooth muscle cells in atherosclerotic lesions and bears significant responsibility in the progression of atherosclerotic plaque (de Souza et al. 2012). Our findings also provided evidence confirmed that macrophage-EV/siHMGB1 distinctly decreased the macrophage infiltration and the formation of foam cells in AS mice. Since the formation of atherosclerotic plaques is associated with the LDL-treated macrophages transforming into foam cells (Tao et al. 2021), we treated macrophages with ox-LDL in our in vitro study. Experimental data has unfolded that macrophages and smooth muscle cells (SMCs) uptake LDL-C in atherosclerotic plaque, leading to plaque formation (Bennett et al. 2016; Barrett 2020). As recently documented, ox-LDL can acetylate HMGB1, further promoting pro-inflammatory cell polarization and recruitment of macrophages (Qi et al. 2021). These results confirmed that silencing of HMGB1 suppressed inflammation in macrophages.

Our work noted that macrophage-EV/siHMGB1 inhibited Caspase-11-dependent macrophage pyroptosis to prevent atherosclerotic plaque formation. In the progression of AS, macrophages and SMCs phagocytose excess lipid and their programmed death results in the formation of necrotic cores (Clarke et al. 2010). Pyroptosis, a newly discovered form of programmed cell death, has been well-characterized to be involved in the pathological process of AS (Chang et al. 2013; Xu et al. 2018; Qian et al. 2021). It has been identified that HMGB1 is capable of activating the noncanonical inflammasome pathway and inducing pyroptosis by delivering LPS and promoting endocytosis (Kim and Kim 2018). Partly in line with our finding, depletion of circPPP1CC relieved Pg-LPS-induced pyroptosis and inflammatory response through down-regulation of HMGB1 (Liu et al. 2021).

It was well known that M1 and M2 macrophages represent two distinct polarized or activated states of macrophages under different stimulating conditions. They possess different functions and phenotypic characteristics to respond to physiological and pathological situations. In atherosclerosis, M1 macrophages exhibit characteristics of inflammation, producing various pro-inflammatory cytokines that contribute to the development of atherosclerosis. In contrast, M2 macrophages possess anti-inflammatory and reparative properties, producing anti-inflammatory cytokines that participate in tissue repair and regeneration (Koelwyn et al. 2018; Yang et al. 2020). Reports have indicated that as a damage-associated molecular pattern (DAMP), HMGB1 translocates to the nucleus in the early stages of liver damage and converts Kupffer cells to the M1 phenotype through stimulation of cell surface receptors such as Toll-like receptors (TLRs), promoting liver inflammation. In the early stages of liver damage, HMGB1 is also activated for autophagic degradation, leading to the conversion of Kupffer cells to the M2 phenotype (Yamate et al. 2023).

Additionally, studies have shown that miR-216a-5p can promote the polarization of M2 macrophages by inhibiting the HMGB1/TLR4/NF-kB signaling pathway (Qian et al. 2023). Furthermore, magnoflorine has been reported to attenuate M1 polarization-induced intervertebral disc degeneration by reducing HMGB1 expression and deactivating the MyD88/NF-kB pathway (Zhao et al. 2021). Moreover, research has revealed that haptoglobin can regulate macrophage/microglial transformation into the M2 phenotype by binding to and clearing HMGB1 (Morimoto et al. 2022). Interestingly, it has been shown that the HMGB1/TLR2 signaling axis is suppressed in M2 macrophages, which may explain the more substantial protective effect of M2 macrophages compared to M1 macrophages in diclofenac-induced hepatotoxicity (Kawase et al. 2022). The articles above collectively demonstrate the regulatory relationship between HMGB1 and M2 macrophages. The reduction of HMGB1, contrary to expectations, leads to an increase in M2 macrophage polarization, which in turn affects tissue inflammation and subsequent phenotypes. Therefore, treatment with oxLDL + MEV/siHMGB1 may paradoxically increase the proportion of M2 macrophages, similar to our findings, and possibly form a negative feedback loop to regulate the occurrence and progression of the disease.

Using the RAW264.7 cell line as a model of macrophages instead of primary bone marrow-derived macrophages may simplify the model and result in the loss of specific physiological characteristics. Despite the wide application and easy accessibility of RAW264.7 cell line in research, its expression profile and function may differ from that of primary macrophages. Andrew Yh Ng et al. conducted a proteomic study comparing these two cell types and discovered significant differences in protein regulation related to cell cycle control, cytoskeletal reorganization, and cell apoptosis. Furthermore, the transformation state of the cells may have a more profound physiological impact on RAW264.7 cells (Ng et al. 2018). However, due to the ease of cultivation and maintenance and the stability under various experimental conditions, the RAW264.7 cell line has been widely applied in studies related to inflammatory response (Nakanishi-Matsui et al. 2012; Hwang et al. 2021). Although we chose the RAW264.7 cell line for our current research, we acknowledge that primary cells are a more physiologically relevant model. Therefore, in future studies, we will consider using primary cells to address atherosclerosis-related issues better.

Many studies have shown that miRNAs derived from macrophage-derived extracellular vesicles (macrophage-EVs) play different roles in the development of atherosclerosis. It has been suggested that macrophage-derived EVs containing mir-199a-5p inhibit the expression of SMARCA4, thereby reducing endothelial cell apoptosis and alleviating atherosclerosis (Liang et al. 2023). In this study, we targeted macrophages with macrophage-EV-loaded siHMGB1 to inhibit the activation of caspase-11 and the extracellular release of HMGB1, thus suppressing macrophage apoptosis and foam cell formation. This is the first report to elucidate the molecular mechanism of macrophage-EV-loaded siHMGB1 in preventing the formation of atherosclerotic plaques, providing a new technical approach and theoretical basis for clinical treatment. This study reveals the potential application of MEV/siHMGB1 in treating inflammatory diseases such as atherosclerosis, opening up new therapeutic avenues. Currently, there are no reports on macrophage-EVs' impact in regulating HMGB1 and the development of atherosclerosis. Understanding the interaction between macrophage-EVs and HMGB1 is crucial in comprehending, preventing, and improving the prognosis of diseases, and it provides a valuable foundation for developing new treatment strategies.

## Conclusions

**Fig. 7:**
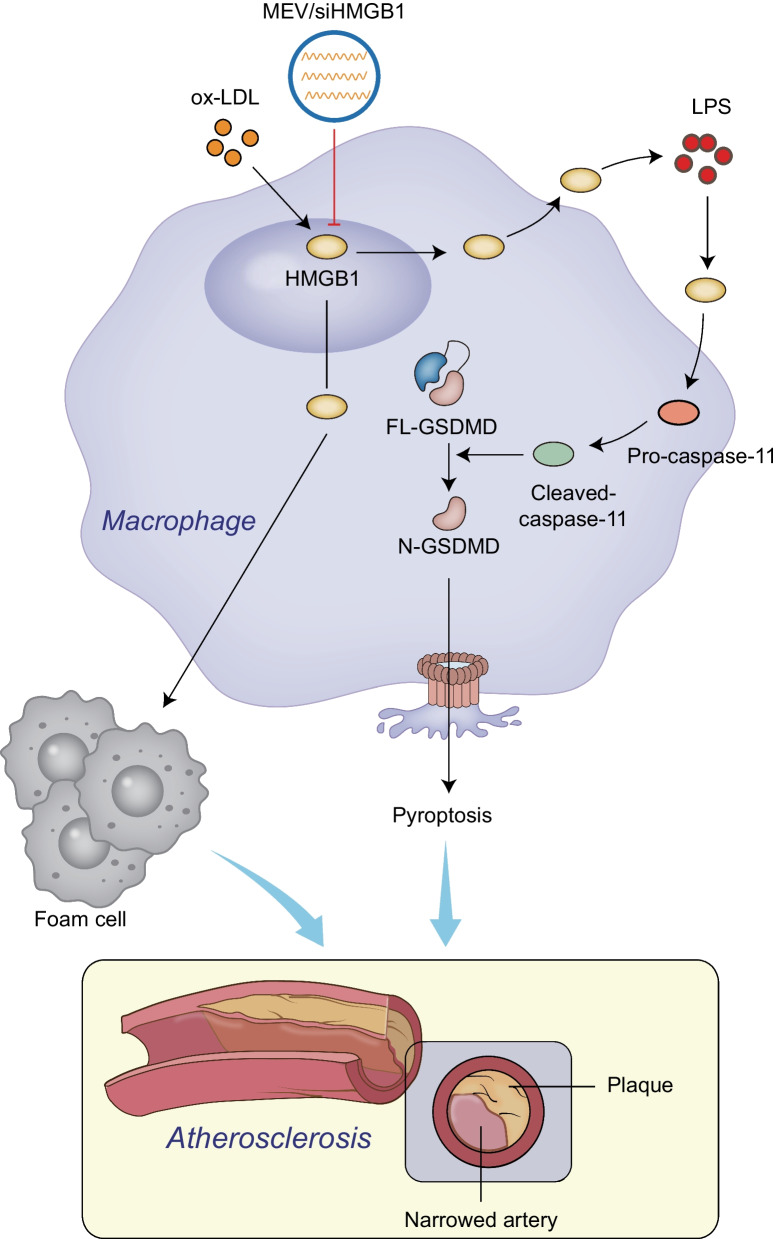
Schematic diagram of the molecular mechanism of macrophage-derived EV-loaded siHMGB1 in atherosclerotic plaque formation. Macrophage-EV/siHMGB1 can inhibit HMGB1 expression in macrophages and reduce its extracellular release, and then inhibit the transformation of macrophages to foam cells, which not only inhibits the activation of Caspase-11 in macrophages but also prevents macrophage pyroptosis, thus restraining atherosclerotic plaque formation

In summary, we have developed a macrophage-EV-based drug-delivery platform, and we concluded that macrophage-EV/siHMGB1 could dampen the formation of plaque in AS and inhibit macrophage pyroptosis, thus reliving AS (Fig. 7). Our results highlighted the therapeutical effect of macrophage-EV/siHMGB1 on AS. Due to its excellent biocompatibility, this technology exhibits potential for further clinical translation.

## Data Availability

The data supporting this study’s findings are available in the manuscript and supplementary materials.

## Abbreviations

AS: Atherosclerosis
LDL: Low-density lipoprotein
oxLDL: Oxidized LDL
macrophage-EVs: Macrophage-derived extracellular vesicles
GEO: Gene Expression Omnibus
GO: Gene Ontology
MF: Molecular function
BP: Biological pathway
CC: Cellular component
PPI: Protein–protein interaction
FBS: Fetal bovine serum
NTA: Nanoparticle tracking analysis
siHMGB1: Small interfering RNA against HMGB1
ELISA: Enzyme-linked immunoassay
IPP: Image Pro Plus
GAPDH: Glyceraldehyde-3-phosphate dehydrogenase
IHC: Immunohistochemistry
IF: Immunofluorescence

## References

[CR1] Abbate A, Toldo S, Marchetti C, Kron J, Van Tassell BW, Dinarello CA (2020). Interleukin-1 and the inflammasome as therapeutic targets in cardiovascular disease. Circ Res.

[CR2] Back M, Yurdagul A, Tabas I, Oorni K, Kovanen PT (2019). Inflammation and its resolution in atherosclerosis: mediators and therapeutic opportunities. Nat Rev Cardiol.

[CR3] Barrett TJ (2020). Macrophages in atherosclerosis regression. Arterioscler Thromb Vasc Biol.

[CR4] Batra R, Suh MK, Carson JS, Dale MA, Meisinger TM, Fitzgerald M (2018). IL-1beta (Interleukin-1beta) and TNF-alpha (Tumor Necrosis Factor-alpha) impact abdominal aortic aneurysm formation by differential effects on macrophage polarization. Arterioscler Thromb Vasc Biol.

[CR5] Bennett MR, Sinha S, Owens GK (2016). Vascular smooth muscle cells in atherosclerosis. Circ Res.

[CR6] Bouchareychas L, Duong P, Covarrubias S, Alsop E, Phu TA, Chung A (2020). Macrophage exosomes resolve atherosclerosis by regulating hematopoiesis and inflammation via MicroRNA cargo. Cell Rep.

[CR7] Burdette BE, Esparza AN, Zhu H, Wang S (2021). Gasdermin D in pyroptosis. Acta Pharm Sin B.

[CR8] Chang W, Lin J, Dong J, Li D (2013). Pyroptosis: an inflammatory cell death implicates in atherosclerosis. Med Hypotheses.

[CR9] Clarke MC, Talib S, Figg NL, Bennett MR (2010). Vascular smooth muscle cell apoptosis induces interleukin-1-directed inflammation: effects of hyperlipidemia-mediated inhibition of phagocytosis. Circ Res.

[CR10] de Souza AW, Westra J, Limburg PC, Bijl M, Kallenberg CG (2012). HMGB1 in vascular diseases: its role in vascular inflammation and atherosclerosis. Autoimmun Rev.

[CR11] de Torre-Minguela C, Barbera-Cremades M, Gomez AI, Martin-Sanchez F, Pelegrin P (2016). Macrophage activation and polarization modify P2X7 receptor secretome influencing the inflammatory process. Sci Rep.

[CR12] Deng M, Tang Y, Li W, Wang X, Zhang R, Zhang X (2018). The endotoxin delivery protein HMGB1 mediates caspase-11-dependent lethality in sepsis. Immunity.

[CR13] Ding JW, Zheng XX, Zhou T, Tong XH, Luo CY, Wang XA (2016). HMGB1Modulates the Treg/Th17 ratio in atherosclerotic patients. J Atheroscler Thromb.

[CR14] Ding L, Zhou W, Zhang J, Tang Q, Xiao W, Chen M (2022). Calming egress of inflammatory monocytes and related septic shock by therapeutic CCR2 silencing using macrophage-derived extracellular vesicles. Nanoscale.

[CR15] EminiVeseli B, Perrotta P, De Meyer GRA, Roth L, Van der Donckt C, Martinet W (2017). Animal models of atherosclerosis. Eur J Pharmacol..

[CR16] Evers MJW, van de Wakker SI, de Groot EM, de Jong OG, Gitz-Francois JJJ, Seinen CS (2022). Functional siRNA delivery by extracellular vesicle-liposome hybrid nanoparticles. Adv Healthc Mater.

[CR17] Feng X, Chen W, Ni X, Little PJ, Xu S, Tang L (2021). Metformin. Macrophage Dysfunction Atheroscler Front Immunol.

[CR18] Gao S, Wang X, Meng LB, Zhang YM, Luo Y, Gong T (2022). Recent progress of chronic stress in the development of atherosclerosis. Oxid Med Cell Longev..

[CR19] Ghaffari S, Jang E, Naderinabi F, Sanwal R, Khosraviani N, Wang C (2021). Endothelial HMGB1 is a critical regulator of LDL transcytosis via an SREBP2-SR-BI axis. Arterioscler Thromb Vasc Biol.

[CR20] Herrington W, Lacey B, Sherliker P, Armitage J, Lewington S (2016). Epidemiology of atherosclerosis and the potential to reduce the global burden of atherothrombotic disease. Circ Res.

[CR21] Hirano S, Zhou Q, Furuyama A, Kanno S (2017). Differential regulation of IL-1beta and IL-6 release in murine macrophages. Inflammation.

[CR22] Hwang SJ, Song YS, Lee HJ (2021). Phaseolin attenuates lipopolysaccharide-induced inflammation in RAW 264.7 cells and zebrafish. Biomedicines..

[CR23] Jiang M, Sun X, Liu S, Tang Y, Shi Y, Bai Y (2021). Caspase-11-Gasdermin D-mediated pyroptosis is involved in the pathogenesis of atherosclerosis. Front Pharmacol..

[CR24] Kake S, Kawaguchi H, Nagasato T, Yamada T, Ito T, Maruyama I (2020). Association between HMGB1 and thrombogenesis in a hyperlipaemia-induced microminipig model of atherosclerosis. In Vivo.

[CR25] Kalinina N, Agrotis A, Antropova Y, DiVitto G, Kanellakis P, Kostolias G (2004). Increased expression of the DNA-binding cytokine HMGB1 in human atherosclerotic lesions: role of activated macrophages and cytokines. Arterioscler Thromb Vasc Biol.

[CR26] Kawase A, Takashima O, Tanaka S, Shimada H, Iwaki M (2022). Diclofenac-induced cytotoxicity in direct and indirect co-culture of HepG2 cells with differentiated THP-1 cells. Int J Mol Sci.

[CR27] Keller M, Ruegg A, Werner S, Beer HD (2008). Active caspase-1 is a regulator of unconventional protein secretion. Cell.

[CR28] Khambu B, Yan S, Huda N, Yin XM (2019). Role of high-mobility group box-1 in liver pathogenesis. Int J Mol Sci.

[CR29] Kim HM, Kim YM (2018). HMGB1: LPS delivery vehicle for Caspase-11-mediated pyroptosis. Immunity.

[CR30] Koelwyn GJ, Corr EM, Erbay E, Moore KJ (2018). Regulation of macrophage immunometabolism in atherosclerosis. Nat Immunol.

[CR31] Liang W, Chen J, Zheng H, Lin A, Li J, Wu W (2023). MiR-199a-5p-containing macrophage-derived extracellular vesicles inhibit SMARCA4 and alleviate atherosclerosis by reducing endothelial cell pyroptosis. Cell Biol Toxicol.

[CR32] Liu M, Yu Y, Jiang H, Zhang L, Zhang PP, Yu P (2013). Simvastatin suppresses vascular inflammation and atherosclerosis in ApoE(−/−) mice by downregulating the HMGB1-RAGE axis. Acta Pharmacol Sin.

[CR33] Liu J, Wang Y, Liao Y, Zhou Y, Zhu J (2021). Circular RNA PPP1CC promotes Porphyromonas gingivalis-lipopolysaccharide-induced pyroptosis of vascular smooth muscle cells by activating the HMGB1/TLR9/AIM2 pathway. J Int Med Res.

[CR34] Liu X, Liu J, Li Y, Zhang H (2022). The correlation between the inflammatory effects of activated macrophages in atherosclerosis and aortic dissection. Ann Vasc Surg..

[CR35] Lu Y, Cui X, Zhang L, Wang X, Xu Y, Qin Z (2022). The functional role of lipoproteins in atherosclerosis: novel directions for diagnosis and targeting therapy. Aging Dis.

[CR36] Mantsounga CS, Lee C, Neverson J, Sharma S, Healy A, Berus JM (2022). Macrophage IL-1beta promotes arteriogenesis by autocrine STAT3- and NF-kappaB-mediated transcription of pro-angiogenic VEGF-A. Cell Rep.

[CR37] Maranto J, Rappaport J, Datta PK (2011). Role of C/EBP-beta, p38 MAPK, and MKK6 in IL-1beta-mediated C3 gene regulation in astrocytes. J Cell Biochem.

[CR38] Moore KJ, Sheedy FJ, Fisher EA (2013). Macrophages in atherosclerosis: a dynamic balance. Nat Rev Immunol.

[CR39] Moreno JA, Sastre C, Madrigal-Matute J, Munoz-Garcia B, Ortega L, Burkly LC (2013). HMGB1 expression and secretion are increased via TWEAK-Fn14 interaction in atherosclerotic plaques and cultured monocytes. Arterioscler Thromb Vasc Biol.

[CR40] Morimoto M, Nakano T, Egashira S, Irie K, Matsuyama K, Wada M (2022). Haptoglobin regulates macrophage/microglia-induced inflammation and prevents ischemic brain damage via binding to HMGB1. J Am Heart Assoc.

[CR41] Naglova H, Bucova M (2012). HMGB1 and its physiological and pathological roles. Bratisl Lek Listy.

[CR42] Nakanishi-Matsui M, Yano S, Matsumoto N, Futai M (2012). Lipopolysaccharide induces multinuclear cell from RAW264.7 line with increased phagocytosis activity. Biochem Biophys Res Commun..

[CR43] Ng AY, Tu C, Shen S, Xu D, Oursler MJ, Qu J (2018). Comparative characterization of osteoclasts derived from murine bone marrow macrophages and RAW 264.7 cells using quantitative proteomics. JBMR plus..

[CR44] Qi X, Wang H, Xia L, Lin R, Li T, Guan C (2021). miR-30b-5p releases HMGB1 via UBE2D2/KAT2B/HMGB1 pathway to promote pro-inflammatory polarization and recruitment of macrophages. Atherosclerosis.

[CR45] Qian Z, Zhao Y, Wan C, Deng Y, Zhuang Y, Xu Y (2021). Pyroptosis in the initiation and progression of atherosclerosis. Front Pharmacol..

[CR46] Qian W, Huang L, Xu Y, Lu W, Wen W, Guo Z (2023). Hypoxic ASCs-derived exosomes attenuate colitis by regulating macrophage polarization via miR-216a-5p/HMGB1 axis. Inflamm Bowel Dis.

[CR47] Tabas I, Garcia-Cardena G, Owens GK (2015). Recent insights into the cellular biology of atherosclerosis. J Cell Biol.

[CR48] Tang L, Chai W, Ye F, Yu Y, Cao L, Yang M (2017). HMGB1 promotes differentiation syndrome by inducing hyperinflammation via MEK/ERK signaling in acute promyelocytic leukemia cells. Oncotarget.

[CR49] Tao J, Qiu J, Lu L, Zhang L, Fu Y, Wang M (2021). ZBTB20 positively regulates oxidative stress, mitochondrial fission, and inflammatory responses of ox-LDL-induced macrophages in atherosclerosis. Oxid Med Cell Longev..

[CR50] Venkatesan B, Valente AJ, Prabhu SD, Shanmugam P, Delafontaine P, Chandrasekar B (2010). EMMPRIN activates multiple transcription factors in cardiomyocytes, and induces interleukin-18 expression via Rac1-dependent PI3K/Akt/IKK/NF-kappaB andMKK7/JNK/AP-1 signaling. J Mol Cell Cardiol.

[CR51] Wan P, Zhang S, Ruan Z, Liu X, Yang G, Jia Y (2022). AP-1 signaling pathway promotes pro-IL-1beta transcription to facilitate NLRP3 inflammasome activation upon influenza A virus infection. Virulence.

[CR52] Wang J, Li P, Xu X, Zhang B, Zhang J (2020). MicroRNA-200a inhibits inflammation and atherosclerotic lesion formation by disrupting EZH2-mediated methylation of STAT3. Front Immunol.

[CR53] Wang Y, Zhang H, Chen Q, Jiao F, Shi C, Pei M (2020). TNF-alpha/HMGB1 inflammation signalling pathway regulates pyroptosis during liver failure and acute kidney injury. Cell Prolif.

[CR54] Wang Y, Zhao M, Liu S, Guo J, Lu Y, Cheng J (2020). Macrophage-derived extracellular vesicles: diverse mediators of pathology and therapeutics in multiple diseases. Cell Death Dis.

[CR55] Willis ML, Mahung C, Wallet SM, Barnett A, Cairns BA, Coleman LG (2022). Plasma extracellular vesicles released after severe burn injury modulate macrophage phenotype and function. J Leukoc Biol.

[CR56] Wu H, Chen Z, Chen JZ, Pei LG, Xie J, Wei ZH (2018). High mobility group B-1 (HMGB-1) promotes apoptosis of macrophage-derived foam cells by inducing endoplasmic reticulum stress. Cell Physiol Biochem.

[CR57] Wu X, Zhang H, Qi W, Zhang Y, Li J, Li Z (2018). Nicotine promotes atherosclerosis via ROS-NLRP3-mediated endothelial cell pyroptosis. Cell Death Dis.

[CR58] Wu G, Zhang J, Zhao Q, Zhuang W, Ding J, Zhang C (2020). Molecularly engineered macrophage-derived exosomes with inflammation tropism and intrinsic heme biosynthesis for atherosclerosis treatment. Angew Chem Int Ed Engl.

[CR59] Xu B, Zhang Y, Du XF, Li J, Zi HX, Bu JW (2017). Neurons secrete miR-132-containing exosomes to regulate brain vascular integrity. Cell Res.

[CR60] Xu YJ, Zheng L, Hu YW, Wang Q (2018). Pyroptosis and its relationship to atherosclerosis. Clin Chim Acta..

[CR61] Yamate J, Izawa T, Kuwamura M (2023). Macrophage pathology in hepatotoxicity. J Toxicol Pathol.

[CR62] Yan G, Chen L, Wang H, Wu S, Li S, Wang X (2021). Baicalin inhibits LPS-induced inflammation in RAW264.7 cells through miR-181b/HMGB1/TRL4/NF-kappaB pathway. Am J Transl Res..

[CR63] Yang S, Yuan HQ, Hao YM, Ren Z, Qu SL, Liu LS (2020). Macrophage polarization in atherosclerosis. Clin Chim Acta..

[CR64] Yang Z, Shi J, Chen L, Fu C, Shi D, Qu H (2022). Role of pyroptosis and ferroptosis in the progression of atherosclerotic plaques. Front Cell Dev Biol..

[CR65] Zhang X, Fernandez-Hernando C (2021). Endothelial HMGB1 (high-mobility group box 1) regulation of LDL (low-density lipoprotein) transcytosis: a novel mechanism of intracellular HMGB1 in atherosclerosis. Arterioscler Thromb Vasc Biol.

[CR66] Zhao F, Guo Z, Hou F, Fan W, Wu B, Qian Z (2021). Magnoflorine Alleviates "M1" polarized macrophage-induced intervertebral disc degeneration through repressing the HMGB1/Myd88/NF-kappaB pathway and NLRP3 inflammasome. Front Pharmacol..

[CR67] Zong P, Feng J, Yue Z, Yu AS, Vacher J, Jellison ER (2022). TRPM2 deficiency in mice protects against atherosclerosis by inhibiting TRPM2-CD36 inflammatory axis in macrophages. Nat Cardiovasc Res.

